# Analysis of Factors Influencing Kidney Function of Recipients After Renal Transplantation in Southwestern China: A Retrospective Study

**DOI:** 10.3389/fmed.2020.519582

**Published:** 2020-11-12

**Authors:** Zhaodan Xin, Lijuan Wu, Juan Zhou, Jie Zhuang, Wu Peng, Turun Song, Tao Lin, Xiaojun Lu, Binwu Ying

**Affiliations:** ^1^Department of Laboratory Medicine, West China Hospital, Sichuan University, Chengdu, China; ^2^West China School of Medicine, Sichuan University, Chengdu, China; ^3^Department of Urology, West China Hospital, Sichuan University, Chengdu, China

**Keywords:** renal transplantation, factors analysis, cystatin C, recipients and donors, short- and long-term influence, kidney function

## Abstract

**Background:** Factors influencing the kidney function of patients after renal transplantation include both recipient-related factors and donor-related factors. To gain a better understanding of these factors and to improve clinical decision-making, we performed a retrospective study of southwestern Chinese people receiving kidney transplantation.

**Methods:** In this retrospective analysis, a total of 2,462 recipients receiving allogeneic kidney transplantation in West China Hospital of Sichuan University from December 13, 2008 to January 10, 2018 were included. Data on recipient and donor characteristics were extracted from the Transplant Center Database and stratified by discrete time points after kidney transplantation. Univariate and multivariate logistic regression analyses were carried out on the study variables, and kidney function of postoperative patients was monitored using cystatin C (CysC) as the outcome indicator.

**Results:** From the univariate analysis, several factors showed statistically significant short-term impact on kidney function based on CysC after kidney transplantation, including age, ethnicity, body mass index (BMI), and HLA A-B-DR-DQ loci mismatch. Gender of recipients and gender-consistency between donors and recipients revealed both short-term and long-term influence. Younger donors had significantly better medium-and-long-term influence on kidney function. From the multivariate logistic regression analysis, recipient gender, ethnicity, BMI, and donor age were independent factors affecting postoperative CysC recovery at discrete time points.

**Conclusion:** Several factors of recipients related to renal function after kidney transplantation, such as gender, ethnicity, BMI and donor's age should be paid more attention to. Moreover, female and non-Han recipients decreased the risk of poor outcome during postoperative kidney function recovery while large BMI of recipients and higher donor age increased the risk. It is useful to predict the postoperative renal function earlier according to corresponding factors, and improve the patient's quality of life.

## Introduction

Chronic kidney disease (CKD) is a great global health burden due to its high associated risk of end-stage renal disease (ESRD) (1) and ESRD has been reported to affect an estimated 7.4 million people worldwide (2). Kidney transplantation has revolutionized the treatment of CKD and is acknowledged as the most effective renal replacement therapy for people with ESRD by improving patient life expectancy and quality of life (3). Owing to its growing population, China has the second highest incidence of kidney transplants in the world, after the United States. Recently, optimizing long-term survival of transplant recipients has become a hot topic in the World Transplantation Congress (4). As a result, in addition to increasing the quantity of kidney transplant procedures, improving the long-term survival rate of recipients and ensuring the quality of renal function after transplantation have become primary objectives for all transplant centers.

To date, numerous studies have demonstrated that many donor- and recipient-related factors are associated with outcomes after kidney transplantation. The outcomes have improved dramatically with the development of matching rules. Baseline demographics such as gender, age, body mass index (BMI), and ABO blood type of donors and recipients have been extensively investigated. Regarding gender, previous work identified sex mismatch to be associated with worse kidney transplant survival outcomes, especially with regards to female donors into male recipients (5), while a more recent study suggests that there is no association between donor-recipient sex mismatch and recipient outcomes (6). Many studies have shown that younger donors result in better outcomes than older donors (7), although some scholars think that renal function can be wasted when kidneys from younger donors are transplanted into older recipients because the recipient may die while the kidney graft is still functional (8). Interestingly, a recent publication by Cohen et al. reported that recipients of offspring donors had higher mortality and graft loss (9). BMI is another important factor affecting transplantation outcomes, as donor obesity is a reported risk factor for graft failure. In contrast, smaller recipients of larger donor kidneys have been linked to better long-term outcomes (10). This difference in BMI can also reduce the possibility of graft loss for sex-mismatched recipients (11). ABO incompatibility has long been regarded as a contraindication to kidney transplantation (12). However, due to increasing organ shortage during the last three decades, ABO incompatibility kidney transplantation has become a routine procedure and death-censored graft survival rates are comparable to those in compatible transplantation (13, 14).

It is clear from the literature that additional research is necessary to determine which factors are most closely related to renal transplantation outcomes and there are still several problems to be addressed. First, analyzing factors univariately without combination may ignore the interaction and confounding impact on outcomes (11). Additionally, research on regional disparity or ethnicity differences is likely to obtain different conclusions. In America, African Americans undergoing deceased donor renal transplantation have lower graft survival compared to Caucasian Americans (15). Similarly, black kidney transplant recipients in the United Kingdom have increased risk of adverse graft-related outcomes owing to high-risk baseline variables (16). While in China there have been few studies analyzing factors of recipients and donors influencing kidney function after transplantation, and no comparisons among diverse ethnic groups (17–21). Therefore, it's high time to conduct corresponding research to provide references for kidney transplantation in China. Additionally, the sample size of each research study has been limited, ranging from 300 to 600 participants (18, 20, 22). However, kidney transplantation is affected by numerous factors, and a larger sample size is required to rule out confounding factors.

To gain a better understanding of the impact of donor- and recipient-related factors on kidney transplantation outcomes and to improve donor candidate evaluation and selection before surgery, we incorporated the effect of multiple factors on kidney function after renal transplantation in a larger population of southwestern Chinese people. To our knowledge, West China Hospital of Sichuan University has done the largest number of kidney transplants in our country, and we retrospectively analyzed data from cases during the past decade. The goal of this study is to acquire information about inferior outcomes earlier to allow clinicians to make appropriate adjustments in therapy at specific time points, thereby enhancing the quality of life of patients.

## Materials and Methods

### Study Population

Our analytic cohort is kidney transplant patients receiving allogeneic kidney transplantation in the Transplant Center Database of West China Hospital of Sichuan University from June 2003 to January 10, 2018 (*N* = 3,067). We subsequently excluded subjects with unknown transplant date (*N* = 3) and those without clinical data on CysC examination and recipient or donor baseline characteristics (*N* = 602). The full analytic cohort contained 2,462 transplant recipients from December 13, 2008 to January 10, 2018. Donors recorded in the Transplant Center Database include immediate or collateral blood-related relative living donors within three generations (*n* = 1,960), as well as, unrelated donors (*n* = 497) who were spouse donors (*n* = 155), a foster father donor (*n* = 1), and donors after cardiac death (DCD) (*n* = 341). All data are anonymous to protect patient privacy. This retrospective study was approved by the Biomedical Ethics Subcommittee of West China Hospital of Sichuan University [reference No. 2015(288)] before subjects participation in the study.

### Study Variables

Donor and recipient characteristics were enrolled and matched in the Transplant Center Database before transplantation. Data extracted from the database for this analysis include: (1) Recipient-related factors: gender, age, BMI (calculated by height and weight), blood type, ethnicity, number of transplants, preoperative hepatitis history, type and duration of dialysis, and HLA A-B-DR-DQ loci mismatch; and (2) Donor-related factors: gender, age, blood type, and relationship to recipient.

### Postoperative Analysis Time Points

Postoperative patients were followed up. According to the conditions of follow-up, the postoperative analysis time points were stratified as 1 day, 1, 2 weeks, 1, 3, 6 months, 1, 2, 3, 4, 5 years, and more than 5 years up to 10 years. However, given the number of patients that were lost to follow-up after 5 years (only 2 patients were followed up for 10 years), the time point of more than 5 years does not appear in the statistical analysis.

### Outcome Indicators

Cystatin C (CysC), also called cystatin 3, is a protein synthesized continuously by nucleated cells. It is under a ubiquitous distribution in human tissue as well as body fluids. CysC is deemed to be a sensitive parameter of the initial renal dysfunction (23). The outcome measurement for this study is the CysC value. The reference range of CysC is 0.51–1.09 mg/L, based on the biochemical laboratory test report of the Laboratory Medicine Department of West China Hospital. Further, the CysC value reflects the estimated glomerular filtration rate (eGFR) and thus shows the kidney condition of postoperative patients. If CysC returned to within baseline, it suggested recovery of kidney function.

### Statistical Analyses

Continuous variables such as age were reported as the mean ± SD (standard deviation), and categorical variables were reported as frequencies (percentages). In univariate analyses, CysC was used as the outcome indicator which was normal and abnormal according to the reference range. The influence of continuous variables such as age on renal function was analyzed by Student's *t*-test, and categorical variables was analyzed by chi-square test. Each factor was statistically analyzed stratified by discrete time points after transplantation. Variables which were statistically significant (*P* < 0.05) in the univariate analyses were entered into the multivariable logistic regression analyses hierarchically by discrete time points. Continuous variables were converted into ordinal categorical variables, and the variable with the smallest value was set as a reference. All analyses were conducted using SPSS software (version 17), with *P* < 0.05 indicating statistical significance throughout.

**Figure 1:**
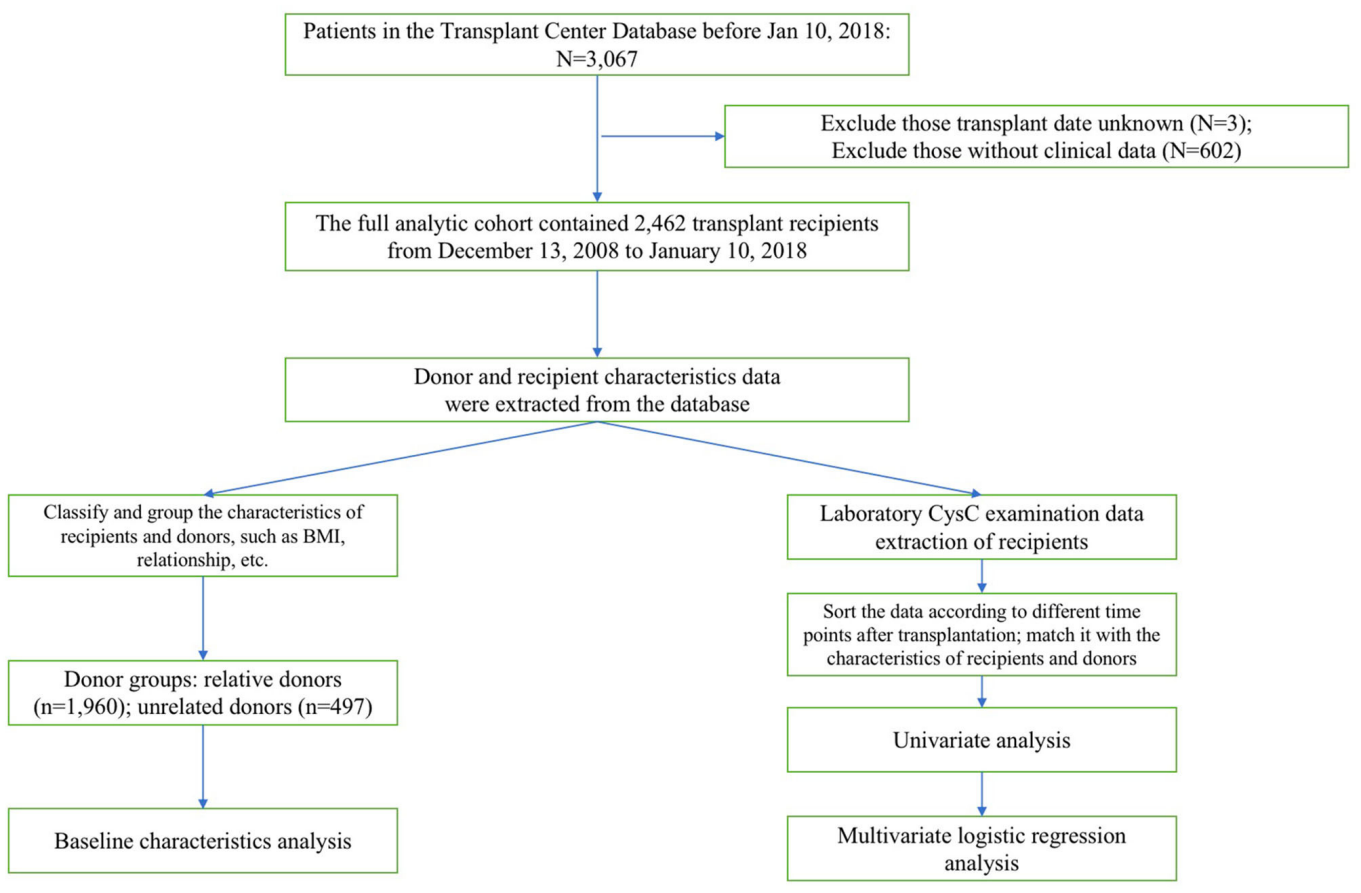
The stepwise approach of the excluded population is detailed in this diagram. Then the subsequent data organization and analysis of included population are demonstrated.

Figure 1 displays the derivation of our research process including the inclusion and exclusion of subjects and the organization and analyses of data.

## Results

### Baseline Characteristics

There were 1,808 male recipients (73.4%). The mean recipient age was 33 years old and the age range was 8–66 years old. The type A blood group was the most frequent (33.5%). Thirty-five recipients (1.4%) received secondary transplantation and 255 recipients (10.4%) had a history of hepatitis before operation. More than half of the recipients (51.9%) spent over 1 year on dialysis and the primary method of dialysis was hemodialysis (90.4%). Recipients with HLA A-B-DR-DQ mismatches less than or equal to 4 accounted for the majority (73.2%).

More donors were females (61.6%) than males. The mean donor age was 46 years old and the age range was 1–68 years old. The type O blood group was the most frequent among the donors, accounting for 46.1%. The relationship between donors and recipients was predominantly relative (79.8%), while unrelated donors, including spouse donors, a foster father and DCD, comprised 20.2% (Table 1).

**Table 1:** Recipient and donor characteristics of the study population (*n* = 2,462).

	**Missing number (*n*)**	**Statistics [N/(percentage)]**
**Recipient parameters**		
Age	0	33.08 ± 9.47
BMI	1,228	21.29 ± 3.74
Gender: male	0	1,808 (73.4)
Female	0	653 (26.5)
Blood type	0	
A type		825 (33.5)
B type		614 (24.9)
O type		778 (31.6)
AB type		245 (10.0)
Ethnicity: Han	26	2,167 (89.0)
Secondary transplantation	0	35 (1.4)
History of hepatitis	10	255 (10.4)
History of dialysis	464	1,998 (81.1)
Hemodialysis	0	1,808 (90.4)
Abdominal dialysis	0	190 (9.6)
Dialysis time >1 year	0	1,037 (51.9)
HLA A-B-DR-DQ loci mismatches	100	
0–4		1,729 (73.2)
5–8		633 (26.8)
**Donor parameters**		
Age	272	46.49 ± 10.94
Gender: male	273	841 (38.4)
Female		1,348 (61.6)
Blood type	192	
A type		648 (28.8)
B type		480 (21.3)
O type		1,036 (46.1)
AB type		85 (3.8)
Relationship	5	
Relative donors		1,960 (79.8)
Unrelated donors		497 (20.2)

### Univariate Analyses

#### Recipient-Related Impact on Postoperative Kidney Function

The age of recipients had a significant effect on kidney function based on CysC from 1 week to 3 months after renal transplantation (*P* < 0.05). Compared against mean age of abnormal CysC recipients, the normal CysC recipients mean age was lower.

Non-Han patients have a faster CysC recovery than Han patients 3 months−2 years after surgery (*P* < 0.05).

The effect of BMI on CysC recovery was significantly different in patients 1 week−2 years after surgery (*P* < 0.05). Smaller BMI recipients had larger percentage of normal CysC.

**Figure 2:**
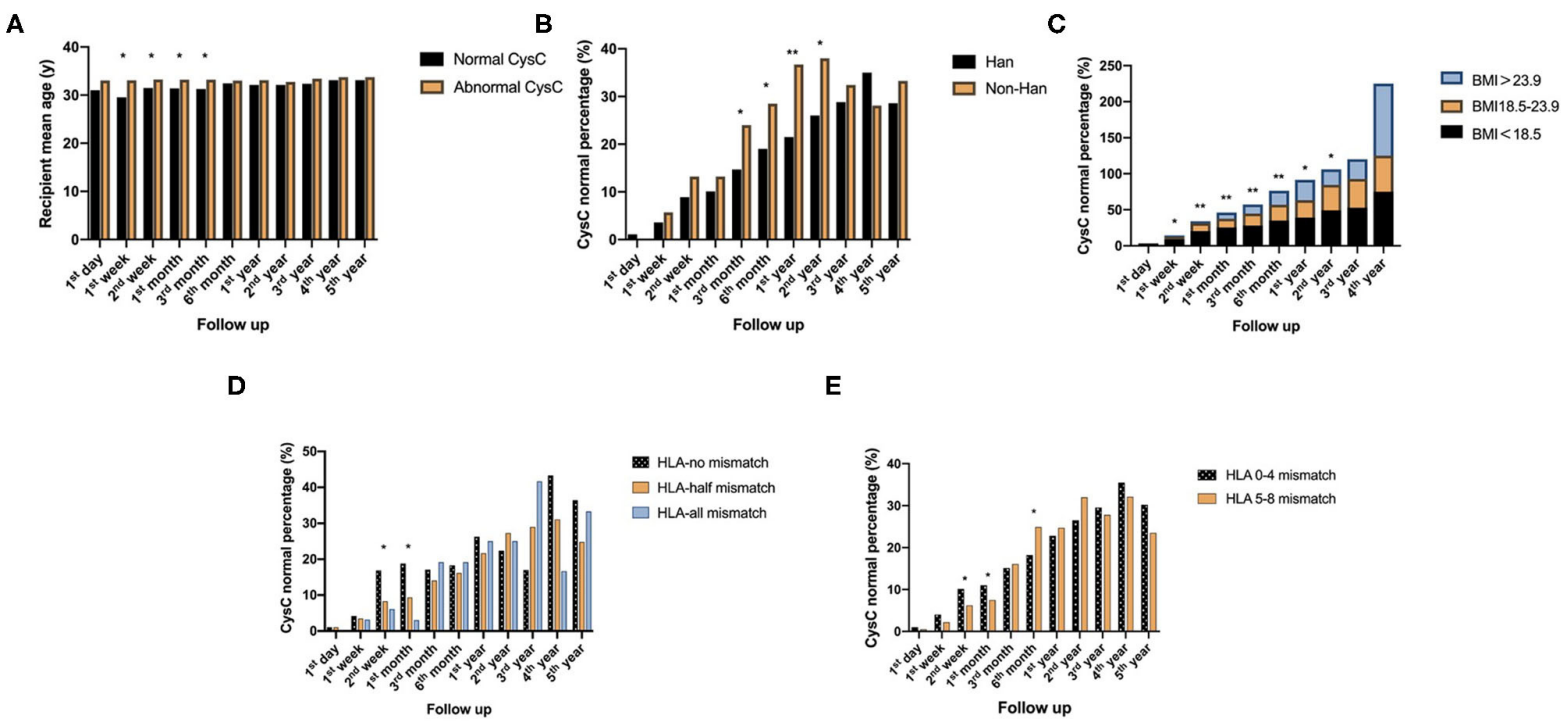
Recipient-related factors of short-term impact on postoperative kidney function reflected by CysC value: **(A)** Comparison of the age of recipients with normal and abnormal CysC at different time points follow up after surgery. **(B)** Comparison of CysC normal percentage between Han and non-Han recipients at different time points follow up after surgery. **(C)** Comparison of CysC normal percentage between groups of different ranges of recipients' BMI at different time points follow up after surgery. **(D,E)** are comparison of CysC normal percentage between different numbers of HLA loci mismatch at different time points follow up after surgery (*P < 0.05; **P < 0.001).

CysC recovery rate was faster 2 weeks−1 month after surgery when the number of HLA A-B-DR-DQ loci mismatches was zero (*P* < 0.05), and compared with the 5–8 mismatch group, the rate of CysC recovery was statistically higher in the 0–4 mismatch group from 2 to 6 months after transplantation (*P* < 0.05), suggesting better recovery of kidney function when there are fewer mismatches (Figure 2).

**Figure 3:**
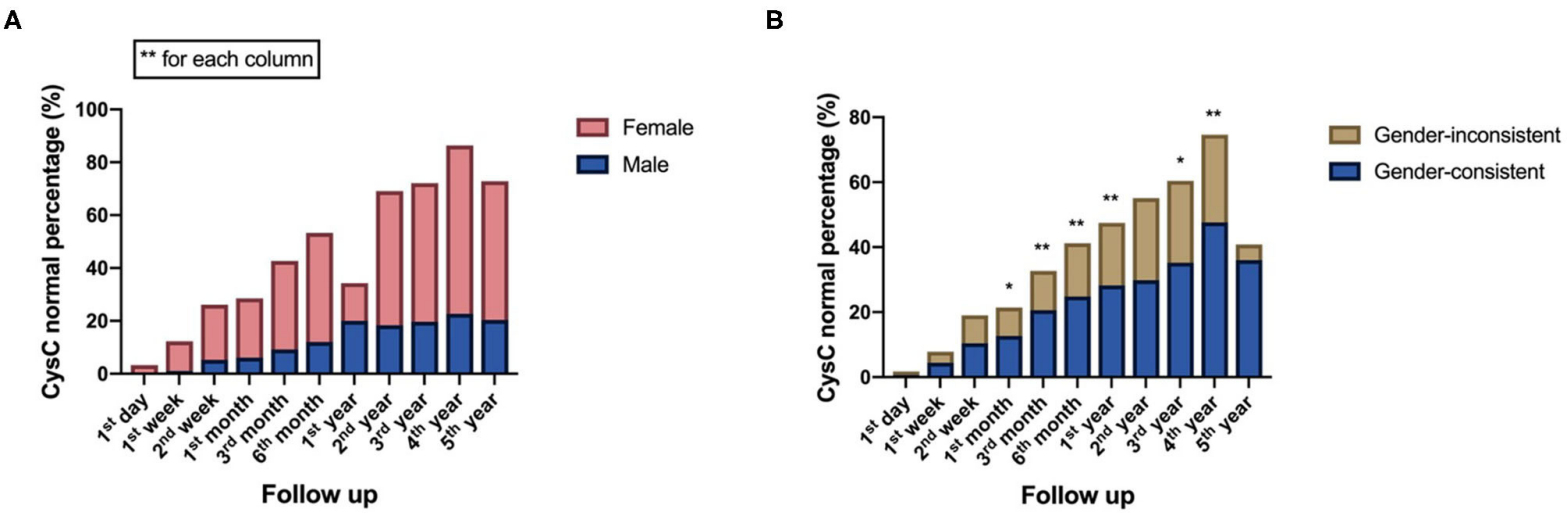
Recipient-related factors of both short-term and long-term impact on postoperative kidney function reflected by CysC value: **(A)** Comparison of CysC normal percentage between male and female recipients at different time points follow up after surgery. **(B)** Comparison of CysC normal percentage between gender-consistent and gender-inconsistent groups of donor-recipient at different time points follow up after surgery (*P < 0.05; **P < 0.001).

There was a statistically significant difference in CysC recovery between female and male recipients from 1 day to 5 years after kidney transplantation (*P* < 0.001), with a faster recovery rate in female recipients. Gender compatible recipients with donors had higher CysC normal percentage from 1 month to 4 years, except at 2 years (*P* < 0.05) (Figure 3).

#### Donor-Related Impact on Postoperative Kidney Function

**Figure 4:**
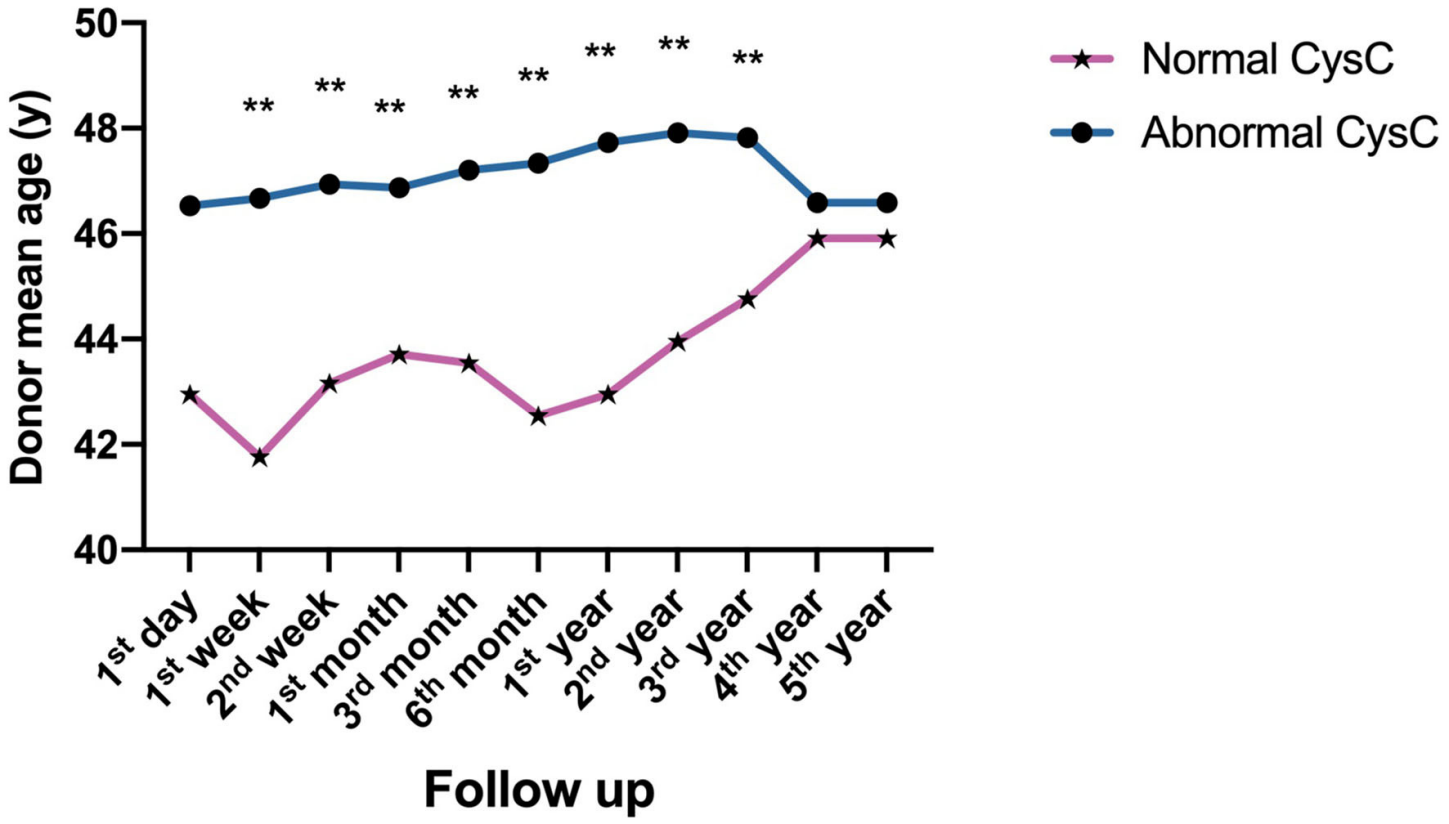
Donor-related factors of impact on postoperative kidney function reflected by CysC value: Comparison of the age of donors with normal and abnormal CysC at different time points follow up after surgery (**P < 0.001).

As for donor-related factors, the recipients receiving a kidney from a younger donor had significantly better impact on kidney function recovery as measured by CysC levels from 1 week to 3 years (*P* < 0.001) (Figure 4).

No statistical significance was found in the influence of preoperative hepatitis history, receiving secondary transplantation, dialysis type and time, blood type compatible/incompatible or relationship between donors and recipients on kidney function (*P* > 0.05).

### Multivariate Logistic Regression Analyses

Statistically significant factors at discrete time point are listed in the Table 2, and their time points are listed beside. The results showed that the recipient gender, ethnicity, BMI, and donor age were independent factors affecting postoperative CysC recovery thus reflecting kidney function in renal transplant recipients (*P* < 0.05). Female gender was a protective factor for CysC recovery from 1 day to 2 years (*P* < 0.05, OR <1), and non-Han minority was a protective factor from 1 week to 3 months (*P* < 0.05, OR <1). While the recipient who received a kidney from an older donor was a risk factor for CysC recovery from 2 weeks to 2 years (*P* < 0.05, OR > 1), as well as the recipients with large BMI from 2 weeks to 1 month (*P* < 0.05, OR > 1).

**Table 2:** Multivariate logistic regression analysis.

**Variables**	**Time point**	**Regression coefficients**	**Wald χ^**2**^-value**	***P*-value**	**OR (95%CI)**
Gender of recipients (Male:1/Female:2)	**1st day**	−3.428	8.411	**0.004**	**0.032 (0.003–0.329)**
	**1st week**	−2.271	21.232	**<0.001**	**0.103 (0.039–0.271)**
	**2nd week**	−1.81	28.866	**<0.001**	**0.164 (0.085–0.317)**
	**1st month**	−1.617	32.492	**<0.001**	**0.199 (0.114–0.346)**
	**3rd month**	−1.49	30.327	**<0.001**	**0.225 (0.133–0.383)**
	**6th month**	−1.689	36.04	**<0.001**	**0.185 (0.106–0.321)**
	**1st year**	−1.694	32.46	**<0.001**	**0.184 (0.103–0.329)**
	**2nd year**	−1.495	10.276	**0.001**	**0.224 (0.09–0.559)**
	3rd year	−0.348	0.484	0.487	0.706 (0.265–1.88)
Donor age grouping [1–35(y):1/36–57(y):2/58–68(y):3]	**2nd week**		9.806	**0.007**	
	*Donor age grouping (1)*	3.18	8.213	**0.004**	**24.041 (2.732–211.546)**
	*Donor age grouping (2)*	2.173	4.372	**0.037**	**8.787 (1.146–67.387)**
	**1st month**		11.818	**0.003**	
	*Donor age grouping (1)*	2.209	11.431	**0.001**	**9.111 (2.531–32.794)**
	*Donor age grouping (2)*	1.269	5.246	**0.022**	**3.557 (1.201–10.537)**
	**3rd month**		11.716	**0.003**	
	*Donor age grouping (1)*	2.429	11.57	**0.001**	**11.351 (2.8–46.023)**
	*Donor age grouping (2)*	1.895	9.264	**0.002**	**6.655 (1.964–22.554)**
	**6th month**		14.869	**0.001**	
	*Donor age grouping (1)*	2.535	14.869	**<0.001**	**12.611 (3.477–45.732)**
	*Donor age grouping (2)*	1.729	9.536	**0.002**	**5.634 (1.881–16.879)**
	**1st year**		10.778	**0.005**	
	*Donor age grouping (1)*	1.791	10.557	**0.001**	**5.998 (2.036–17.673)**
	*Donor age grouping (2)*	0.714	3.077	0.079	2.041 (0.92–4.531)
	**2nd year**		10.462	**0.005**	
	*Donor age grouping (1)*	2.818	9.037	**0.003**	**16.736 (2.666–105.066)**
	*Donor age grouping (2)*	2.019	8.588	**0.003**	**7.528 (1.951–29.043)**
	3rd year		4.257	0.119	
	*Donor age grouping (1)*	1.671	2.839	0.092	5.315 (0.761–37.116)
	*Donor age grouping (2)*	1.155	3.627	0.057	3.175 (0.967–10.427)
Ethnicity (Han:1/Non-Han:2)	**1st week**	−1.122	5.288	**0.021**	**0.326 (0.125–0.847)**
	**3rd month**	−0.641	3.937	**0.047**	**0.527 (0.28–0.992)**
BMI (<18.5:1/18.5–23.9:2/>23.9:3)	**2nd week**		4.898	0.086	
	*BMI (1)*	1.792	4.757	**0.029**	**6 (1.199–30.021)**
	*BMI (2)*	1.614	4.466	**0.035**	**5.025 (1.124–22.46)**
	**1st month**		5.704	0.058	
	*BMI (1)*	1.276	5.405	**0.02**	**3.581 (1.222–10.497)**
	*BMI (2)*	0.765	2.466	0.116	2.148 (0.827–5.58)

*p < 0.05 indicates statistical significance. If p < 0.05, the corresponding Time Point and OR (95%CI) are highlighted in bold*.

## Discussion

This retrospective study incorporated 2,462 individuals undergoing renal transplantation during the last decade in southwestern China. We identified recipient-related and donor-related factors associated with influence on postoperative kidney function in kidney transplant recipients. From the univariate analyses, we observed that recipient-related factors such as age, ethnicity, BMI, and HLA loci mismatch were related to short-term influence on kidney function after renal transplantation; specifically, the gender of recipients as well as gender-consistency were strongly associated with both short-term and long-term postoperative kidney function. In addition, the age of donors correlated with medium-and-long-term impact on kidney function. Based on the multivariate logistic regression analyses, we found that gender, ethnicity, BMI of recipients, and age of donors were independent influence factors. More specifically, being female and non-Han were protective factors whereas having a large BMI and or an older donor were risk factors for poor postoperative kidney function within 2 years.

Multivariate logistic regression analysis avoids the confounding factors so the independent factors are clear. In our study, we identified female and non-Han recipients decreased the risk of poor outcome during postoperative kidney function recovery while larger BMI of recipients and higher donor age increased the likelihood. However, these results were not totally consistent with Dunn et al. (24), whose risk factors included female recipients. It is possible that the largely Caucasian population in their research showed different general genetic influences and immunological mechanisms compared with our cohort population. Besides, there are many potential differences between diverse population such as socioeconomics, pharmacogenomics, social systems, access to care that may explain the disparate outcomes (25).

In our study, female recipients had better kidney function than male ones. This is understandable because BMI has a significant impact on gender-dependent responses to renal transplantation. A higher BMI means greater demand on the kidney, that is, female recipients need fewer nephrons leading to a better outcome. Besides BMI, hormonal circumstances may also have an impact. Experimental evidence is compatible with the hypothesis that estradiol is protective against the progression of chronic rejection in rat renal transplantation (26). More evidence shows that testosterone and estrogens have contrasting effects on chronic allograft nephropathy. Testosterone promotes allograft injury, whereas the administration of estradiol improves graft function (27).

Transplantation kidney function recovery in non-Han vs. Han Chinese individuals has not previously been investigated in China, but our results show that non-Han recipients have a better graft condition after kidney transplantation than Han recipients. This difference may be related to the physical constitution and living habits of different ethnic groups, but this hypothesis has yet to be explored. Nevertheless, one limitation of our study is that the various minorities were not studied separately to determine which minority may have the best graft condition because the sample size available for separate analysis of each national minority was small. Another one is that we are unable to compare the differences in posttransplant kidney function in the four groups i.e., non-Han recipient/Han donor etc. for not knowing the ethnicity of donors. In the future, we will collect the basic information of donors as completely as possible to investigate the influence of donor factors on transplantation kidney function.

Our study, in accordance with previous research (28), found that high BMI was a risk factor for poor short-term CysC recovery. It has been reported that large recipients are worse with small donors. Among the immune-independent factors is a possible role for inadequate nephron dosing leading to glomerular hyperfiltration and progressive graft damage (29).

When examining donor-related factors, we found that recipients of kidneys from younger donors had better kidney function, possibly because these donor allografts were less likely to suffer graft dysfunction (30). High donor age may lead to an early reduction in the number of functioning nephrons. An imbalance between the nephron mass of the graft and the recipient's need will impair the prognosis of the organ.

Not surprisingly, younger recipients had better postoperative kidney function in our study though without statistical significance in the multivariate analysis. Consistent with our results, several studies in the literature have shown a strong association between age and adverse effect in kidney transplantation (31, 32). This study adds to the literature by analyzing a large cohort of recipients in southwestern China. Younger recipients may have stronger immune systems and higher functioning nephrons, leading to faster recovery. This may also explain why the recipient age is associated with short-term influence postoperatively in our study.

Also as expected, fewer HLA loci mismatches led to better postoperative kidney function in our study in the univariate analysis. This is consistent with earlier studies (20, 33) and has great clinical value. It has also been reported that live-donor transplantation after desensitization provided a significant survival benefit for patients with HLA sensitization, as compared to waiting for a compatible organ (34). In our study, kidneys were mainly from living donors which may be beneficial to better outcomes of recipients. However, in our study, HLA loci mismatch was only found to be associated with short-term influence after transplantation. When there are HLA mismatches, requirement for higher immunosuppression doses and more antirejection therapy are needed, thus it is associated with short-term adverse function because of infection or cardiovascular disease during the first year after kidney transplantation, which persisted but was far less marked during years 2–5 (35). Additionally, in HLA incompatible transplantation, pre-formed donor-specific HLA antibodies may have been present for many years before the transplant, and the level in the early post-transplant period may show large changes. The antibody response may evolve into acute clinical rejection leading to short-term poor kidney function (36).

Interestingly, in univariate analysis, we found no significant effect for matching blood group between donor and recipient, which was not consistent with clinical practice and previous knowledge. However, some studies have reported an additional 10–20% of living donor procedures were conducted by using ABO incompatible kidney transplantation (37, 38). We considered that this was due to the preventive immunosuppression therapy given to transplant recipients to reduce the risk of an undesirable outcome, but more in-depth exploration was necessary. As reported clinically, the desensitization protocols can reduce and maintain anti-A/B antibodies (isoagglutinins) during the first 2 weeks after transplantation below a threshold that is thought to be safe (e.g., <1:32 in tube technique). Thereafter, even when anti-A/B antibodies recur at high levels they will not harm the kidney transplant, a phenomenon that is called accommodation (13). Additionally, the frequency of blood group O significantly increased in the donor population compared with the recipients in our study. This was linked to our kidney allocation principle. The principle of kidney allocation was based on same blood type match priority as well as HLA matching criteria, which was synthetically considered. Not until the matching of the same blood type was unsuccessful, we considered the allocation of different but compatible blood types, that is, the O-type donor to the non-O-type recipient in the next place. Consequently, it led to a higher proportion of O blood type among donors. Compatible ABO blood type was preferred, but when the matching was unsuccessful, the incompatible ABO kidney transplant was considered for reducing waiting time when kidney donors were extremely lacking. Based on these results, it is possible to expand the living donor pool for patients which may reduce waiting time and minimize suffering before transplantation.

Moreover, in our research, most patients received kidney transplantation from their relatives. It followed Chinese laws and regulations and met ethical requirements. More importantly, it had the advantages of better kidney quality, good HLA matching, short donor kidney ischemia time, and selective operation.

Different from previous research, this study used a laboratory indicator CysC, which was not subject to gender, age, or weight (39), to predict renal transplantation kidney function outcomes. Moreover, we incorporated time-hierarchical analysis to discover which postoperative time point was most critical. Previously the “gold standard” diagnostic tool to assess graft failure was kidney biopsy. While this method can precisely determine the condition of the graft and provide direct evidence for surgeons, it remains invasive and expensive, and is a complicated means to obtain samples. Creatinine (Cr) has served as a marker of renal function for several decades. However, this marker has several well-known limitations such as dependent on a number of factors including tubular resorption, total muscle mass, nutritional status, age and gender. Creatinine-based equations for estimation of GFR (eGFR) were established to overcome some of these inadequacies (40). However, estimation equations are reliable only in stable conditions and should not be used when rapid changes of renal function occur (41). Assessment of GFR also requires a 24 h urine collection which is not convenient and may be subject to additional preanalytical errors. For these reasons, graft rejection or chronic allograft dysfunction is usually discovered too late to adjust therapeutic measures (15). CysC, a cysteine protease inhibitor, possesses many attributes required for the ideal eGFR marker. Visvardis et al. indicated that measurement of serum CysC was a useful and accurate surrogate marker for eGFR in renal transplant patients (16). Additionally, measuring CysC is an inexpensive and convenient method to determine renal function, it can be used as an sensitive early warning system for graft failure after transplantation. Determination of CysC can diagnose early-stage renal dysfunction and monitor renal function over time (23). CysC is a better estimate of GFR than Cr, particularly to detect minor GFR reductions, because the variation of Cr rises only when the kidney function is already significantly impaired. Therefore, we utilized CysC as postoperative outcome indicator rather than the traditional kidney biopsy and traditional laboratory indicators.

In the study of correlated factors of kidney transplant patients in China, the sample size of our study is very large. There are, however, some limitations in our study. First, though the quantity of samples is large, this study is only a single-center retrospective analysis, certain restrictions not only in the region but also in the time period still exist. Multi-center data analysis would be an optimal next step to confirm the results. Another potential weakness of this study is that too many transplant recipients were lost to follow-up after 5 years, which mainly due to their return to local hospitals for examination and treatment, so no data were analyzed beyond this time point. Future research will ideally collect data 10 years or more after surgery. We will collect correspondingly comprehensive data for more exhaustive analysis in the future if possible.

In conclusion, our results provide contemporary data on factors influencing both short- and long-term kidney function outcomes after kidney transplantation measured by sensitive parameter CysC. These results not only help clinicians to draw attention to the postoperative renal function in time according to the preoperative factors of patients, but also expand donor screening and select optimal kidney donors.

## Data Availability Statement

The datasets analyzed in this article are anonymous to protect patient privacy and are not publicly available. Request to access the datasets should be directed to email the corresponding author.

## Ethics Statement

The studies involving human participants were reviewed and approved by the Biomedical Ethics Subcommittee of West China Hospital of Sichuan University [reference No. 2015(288)]. Written informed consent from the participants' legal guardian/next of kin was not required to participate in this study in accordance with the national legislation and the institutional requirements.

## Author Contributions

ZX and LW searched the literature, performed the research, wrote, and revised the manuscript. JZhu, WP, and TS provided expert advice and assisted data collecting. ZX, JZho, and TL participated in the analysis of data. ZX, LW, and XL engaged in the acquisition of data (laboratory or clinical). XL and BY designed the study and gave administrative support. All authors contributed to the article and approved the submitted version.

## Conflict of Interest

The authors declare that the research was conducted in the absence of any commercial or financial relationships that could be construed as a potential conflict of interest.

## Abbreviations

CKD: chronic kidney disease
ESRD: end-stage renal disease
CysC: cystatin C
DCD: donor after cardiac death
eGFR: estimated glomerular filtration rate
Cr: creatinine
BMI: body mass index.
